# A feasibility analysis of the ArcBlate MR-guided high-intensity focused ultrasound system for the ablation of uterine fibroids

**DOI:** 10.1007/s00261-021-03203-8

**Published:** 2021-07-09

**Authors:** Chin-Jung Wang, Gigin Lin, Yi-Ting Huang, Cindy Hsuan Weng, Kai-Yun Wu, Yu-Ying Su, Yu-Shan Lin, Kit-Sum Mak

**Affiliations:** 1 Division of Gynecologic Endoscopy, Department of Obstetrics and Gynecology, Chang Gung Memorial Hospital at Linkou, Taoyuan, Taiwan; 2 Chang Gung University College of Medicine, Taoyuan, Taiwan; 3 Department of Medical Imaging and Intervention, Chang Gung Memorial Hospital at Linkou, Taoyuan, Taiwan

**Keywords:** Uterine fibroid, Leiomyoma, Magnetic resonance-guided high-intensity focused ultrasound

## Abstract

**Purpose:**

Uterine fibroids are benign gynecologic tumors and commonly occur in women by the age of 50. Women with symptomatic uterine fibroids generally receive surgical intervention, while they do not favor the invasive therapies. To evaluate the feasibility and safety of a novel magnetic resonance-guided high-intensity focused ultrasound (MRgHIFU) modality, ArcBlate, in the treatment of uterine fibroids.

**Methods:**

Nine patients with uterine fibroids and one patient with adenomyosis were treated with ArcBlate MRgHIFU. Tumor size and quality of life were evaluated postoperatively at 1 and 3 months by magnetic resonance imaging (MRI) and the 36-Item Short Form Survey (SF-36), respectively.

**Results:**

All patients completed the ArcBlate MRgHIFU procedure and there were no treatment-related adverse effects either during the procedure or during the 3 months of follow-up. Despite limiting the ablation volume to under 50% of the treated fibroid volume as a safety precaution, tumor volumes were markedly reduced in four patients by 15.78–58.87% at 3-month post-treatment. Moreover, SF-36 scale scores had improved at 3 months from baseline by 2–8 points in six patients, indicating relief of symptoms and improved quality of life.

**Conclusion:**

This study evidence demonstrates the safety and feasibility of ArcBlate MRgHIFU and suggests its potential for treating uterine fibroids.

## Introduction

Uterine fibroids are benign gynecologic tumors found in the uterus [1, 2] and are the most common of all solid pelvic tumors, affecting 70% of women by age 50 years [3]. Many women with uterine fibroids are asymptomatic and underdiagnosed [4, 5], while in those who are symptomatic, the fibroid growth may compress the endometrium, nerves, and surrounding organs, causing discomfort, inconvenience [6, 7], and impaired quality of life [8, 9]. Tumors may also cause infertility, pregnancy loss, and placental abruption.

Symptomatic uterine fibroids generally require surgical intervention, such as hysterectomy and myomectomy [10]. However, these invasive treatments are not popular choices among women, who often delay treatment by 5 years on average, and prefer uterine-conserving procedures [9]. Non-invasive high-intensity focused ultrasound (HIFU) ablation has emerged as an alternative option to surgery for uterine fibroids, offering minimal invasiveness without an incision that can be performed as a uterine-conserving outpatient treatment in most types of uterine fibroids [11–14]. Compared to the uterine artery embolization, another minimal-invasive treatment option, HIFU is preferred if clinicians and patients have concerns for low vascularity fibroids, high risk for anesthesia/sedation, radiation exposure, impaired renal function, allergy to iodine contrast medium, bleeding tendency, or difficult vascular access [15–17]. Localized ablation is achieved by concentrating acoustic waves at a focal point to cause heat and raise focal temperature to over 55 °C, without affecting the surrounding tissues [18, 19]. This energy transmission causes coagulative necrosis, resulting in resorption and shrinkage of the ablated tumor. Two well-known image guidance methods are coupled with the HIFU system; ultrasound guidance (USgHIFU) and magnetic resonance guidance (MRgHIFU). Several USgHIFU systems are commercially available, but their widespread adoption is limited by the lack of thermal monitoring and high-resolution images for precise positioning. The real-time thermal monitoring and high-quality imaging provided by MRgHIFU ensures its superiority over USgHIFU, but MRgHIFU may be limited by the operation and specific requirements of different MRI systems. Another limitation of most marketed HIFU systems is that they require patients to remain prone for several hours during the procedure, which is not only uncomfortable for them but also increases their risk of skin burns.

The novel MRgHIFU system (the ArcBlate focused ultrasound ablation system) addresses these limitations by combining an arc-shaped treatment machine (an upgrade of the present MRI table) with an automated 3-dimensional navigation along the patient’s abdomen in the supine position and an intuitive *graphical user interface (*GUI) real-time thermal monitoring tool. This study planned to evaluate the feasibility and safety of the ArcBlate focused ultrasound ablation system for the treatment of uterine fibroids.

## Materials and methods

This study was approved by Taiwan’s Food and Drug Administration (TFDA) and the Institutional Review Board of BLINDED INFORMATION (BLINDED INFORMATION; reference number: 104-4660A) and was conducted in accordance with the Declaration of Helsinki Ethical Principles and Good Clinical Practices. Each subject gave a written informed consent form. The datasets used and analyzed during the current study were available from the corresponding author on reasonable request.

### ArcBlate focused ultrasound ablation system

The first prototype of the ArcBlate focused ultrasound ablation system was developed by the Department of Biomedical Engineering in the National Health Research Institute (NHRI), which initially tested the prototype in 7 mini pigs between 2009 and 2011, then conducted a pilot study involving 6 patients with uterine fibroids in BLINDED INFORMATION in 2015 (NCT02283502). EpiSonica undertook subsequent development and manufacture from 2014 and finished this study.

**Fig. 1:**
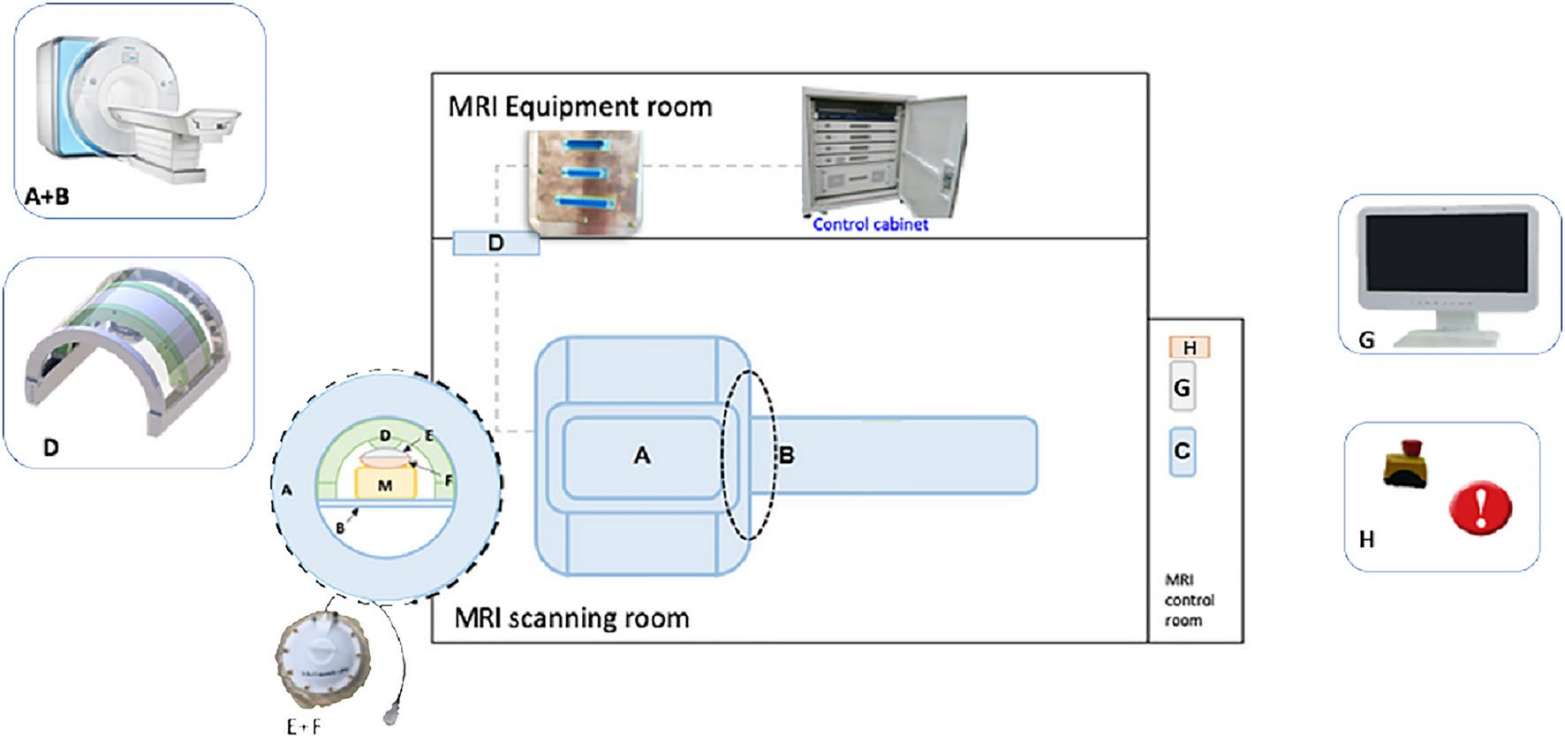
Placement of the ArcBlate system. **A** MRI. **B** Patient table. **C** MRI console. **D** Portable ARC. **E**, **F** HIFU transducer and the water bag. **G** HIFU console. **H** Emergency stop

In contrast to other commercially available MRgHIFU systems, the ArcBlate focused ultrasound ablation system (100 M, manufactured by EpiSonica Corporation, Hsinchu, Taiwan) consists of three parts; a special portable arc for anchoring the ultrasound detector, a control cabinet, and a control console. Figure 1 shows the placement of the ArcBlate system. The portable ARC is designed for easy attachment to the MR patient table and can perform a three-dimensional movement of 20 cm horizontally along the patient table (longitudinal axis), allowing for a ≤ 20° shift along the arc (horizontal axis) and ≤ 20° rotation, in combination with a one-dimensional ArcBlate transducer to allow vertical movements to depths of 6–20 cm. A 15-channel annular array transducer integrated with a flexible water bag was designed for mounting on the portable ARC for generating high-intensity focused ultrasound energy. The flexible water bag is filled with degassed water and is in direct contact with the patient’s skin to enable propagation of the ultrasound beam to the target tumor. The positioning of the portable ARC and the ultrasonic energy delivered by the HIFU transducer is controlled by the console through the control cabinet.

### Patients

Ten patients, including nine with uterine fibroids and one with adenomyosis, were enrolled. Inclusion criteria were (1) benign uterine tumor, such as uterine fibroids, adenomyoma, or adenomyosis; (2) tumor size between 4 and 15 cm or an adenomyosis area larger than 4 cm; (3) age > 20 years and scheduled for a hysterectomy; (4) abdominal circuit under 100 cm. Exclusion criteria were (1) pregnancy; (2) hemoglobin below 9 g/dL; (3) other pelvic diseases; (4) unsuitable HIFU beam path; (5) MRI contraindications; (6) medical history of calcified gynecologic tumor; (7) co-existing diseases including heart, vascular, or renal; (8) malignant uterine tumors identified by MRI; (9) failure to satisfy study enrollment criteria after an assessment by an expert gynecologist and/or radiologist.

The diagnostic criteria for adenomyosis using MRI included (1) low signal intensity of myometrium, with indistinct margins and (2) diffuse or focal junctional zone exceeding 12 mm [20].

### Pretreatment screening and preparation

Before treatment, all subjects underwent routine blood testing and quality of life assessment by the 36-Item Short Form 36 Health Survey (SF-36) questionnaire. MRI was performed within 30 days of these testing procedures using the 3 T Trio MRI scanner (Siemens Healthcare, Erlangen, Germany).

Standard T2-weighted imaging using three orthogonal planes (i.e., coronal, sagittal, and transverse) assigned the Funaki classification and evaluated the size, volume, location, and status of the benign uterine tumor. Standard T1-weighted imaging identified the benign or malignant uterine tumor after the intravenous administration of a gadolinium-based contrast agent (Magnevist, Bayer Pharma AG, Berlin, Germany; dose: 0.2 mL/kg). Other pre-procedural preparations included an 8-h fasting period and enema administrations to exclude air and other possible obstacles (e.g., bowel loops anterior to the uterus), and abdominal and pubic hair shaving to allow the skin to connect with the ultrasound beam closely to prevent the skin burn [21–23]. Bladder filling was conducted only if the fibroid location was close to the intestinal tract.

### MRgHIFU treatment

The patient was placed on the MRI table in the supine position. The portable ARC was put on the MRI table and the water bag was placed in direct contact with the patient’s abdominal skin. Sterile ultrasound gel was applied to the contact area to eliminate air gaps between the water bag and the patient’s skin surface. A quick T2-weighted MR image in the sagittal plane was acquired to confirm the absence of air bubbles between the water bag and the patient’s skin surface.

Subsequently, high-resolution MR images in three orthogonal planes for treatment planning were acquired using a T2-weighted turbo-spin echo sequence with the following parameters: variable TR depending on different planes and/or different patients, TE = 120 ms, flip angle = 120 degrees, matrix size = 512 × 358, field of view (FOV) = 384 × 384 mm^2^, and slice thickness = 4 mm without gaps. These MR images were displayed on the ArcBlate user control GUI, software contained inside the control console designed to assist the physician with establishing the treatment plan based on the tumor characteristics and to identify critical organs such as the bladder, bowel, and spine surrounding the targeted fibroids.

At the treatment planning stage, a single-point treatment pattern, six square-shaped treatment patterns consisting of treatment points (i.e., 4 and 9 points), and gap distances (i.e., 3, 6, or 10 mm gap) were manually selected and inserted in the coronal plane of the T2-weighted MR images. These different treatment patterns helped the physician establish the treatment plan for each patient. Moreover, the power (i.e., 120–300 W), ablation time (i.e., 10–60 s), and cooling time (i.e., 5–60 s) were defined for each treatment pattern. Based on the individual variability, several test sonications with low energy using a single-point treatment pattern were performed in the center of the targeted fibroid on the deeper MR coronal plane to verify the location of heating and to determine the power level for ablation. The treatment plan for each patient was established slice-by-slice from a deeper MR coronal plane, and the planned treatment pattern in each slice was put within a 10-mm distance away from the margin of the targeted fibroid. The planned treatment tumor volume was up to 50% of the fibroid according to safety considerations defined by the TFDA and treatment time was kept to within 1–2 h.

The treatment dose was measured with the following formula:

$${\text{Treatment~dose~}}\left( J \right) = \sum {\text{Power}}\left( W \right) \times {\text{Number}}\;{\text{of}}\;{\text{treatment}}\;{\text{points}}\;{\text{in}}\;{\text{a}}\;{\text{treatment}}\;{\text{pattern}} \times {\text{Ablation}}\;{\text{time}}\;\left( {\sec } \right)$$

During the treatment, the ArcBlate transducer was mounted under the portable ARC and automatically moved to the planned treatment location mapped out in the patient’s treatment plan. Real-time thermal monitoring was also performed in three adjacent coronal slices, using a fast spoiled gradient-recalled-echo sequence with the following parameters: TR = 11 ms, TE = 7 ms, flip angle = 30 degrees, matrix size = 128 × 128, FOV = 384 × 384 mm^2^, slice thickness = 4 mm with a 4 mm gap, number of slices = 3 with the center slice placed at the planned treatment plane, and temporal resolution = 4.2 s/dynamic. Following thermal imaging scanning, 3 dynamics were performed and then the sonications began. Thermal imaging continued to scan more than 10 dynamics after the sonication stopped. These temperature maps were calculated from raw data centered in the k-space matrix and based on the proton resonance frequency shift [24, 25], and then displayed on the ArcBlate user control GUI to assist the physician to monitor temperature changes in the focal, near, and far fields along the ultrasound beam axis.

No patients required sedation, anesthesia, a urinary catheter, or MRI contrast agent. At the end of the treatment, patients were monitored for at least one hour for the occurrence of any acute adverse events.

### Post-treatment follow-up

After discharge, patients were followed-up by a telephone call at 1 week for the assessment of postoperative adverse events. At 1 and 3 months after treatment, quality of life was assessed with the SF-36 questionnaire and tumor volume was determined by MRI.

### Assessments and data analysis

Classification of the benign uterine tumor by fibroid (i.e., subserosal, intramural, submucosal, or adenomyosis) and Funaki type was based on the signal intensity of the T2-weighted MR images [26], in the judgment of one of the investigators (G.G.L., with 15 years of experience in analyzing gynecological MRI images). The Funaki classification evaluates uterine fibroids as either type 1 (low; comparable to that of skeletal muscle), type 2 (intermediate; lower than that of the myometrium and higher than that of the skeletal muscle), or type 3 (high; equal to or higher than that of the myometrium). This classification has been adopted in clinical practices at most institutions performing MR-guided HIFU ablation.

Tumor volume was calculated with the following formula:

$${\text{Tumor}}\;{\text{volume~}}\left( {{\text{cm}}^{3} } \right) = \frac{1}{6}\pi \cdot d_{1} \left( {{\text{cm}}} \right) \cdot d_{2} \left( {{\text{cm}}} \right) \cdot d_{3} \left( {{\text{cm}}} \right)$$

where $\pi$ equals 3.14, and ${d}_{1}$, ${d}_{2}$, and ${d}_{3}$ equal the longest diameter in three orthogonal planes (i.e., coronal, sagittal, and transverse) of the benign uterine tumor measured on the T2-weighted MR images. Treatment effect was measured by fibroid shrinkage using T2-weighted MR images.

Shrinkage of the tumor volume was calculated with the following formula:

$${\text{Shrinkage}}~\left( \% \right) = \frac{{{\text{Tumor}}\;{\text{volume}}\;{\text{after}}\;{\text{treatment}}\left( {{\text{cm}}^{3} } \right) - {\text{Tumor}}\;{\text{volume}}\;{\text{before}}\;{\text{volume}}\left( {{\text{cm}}^{3} } \right)}}{{{\text{Tumor}}\;{\text{volume}}\;{\text{before}}\;{\text{treatment}}~\left( {{\text{cm}}^{3} } \right)}} \times 100$$

The SF-36 questionnaire was used for evaluating quality of life as described in the previous research. Higher scores indicated better quality of life [27].

## Results

Ten patients were enrolled in this study. The median age was 44 years (range 34–49 years). Of them, four had subserosal myomas, three had intramural myomas, two had submucosal myomas, and one had diffuse adenomyosis. The fibroids were classified as Funaki type 1 (*n* = 4), type 2 (*n* = 3), and type 3 (*n* = 2). Before treatment, the mean fibroid volume was 273.4 ± 32.10 cm^3^ for subserosal myomas, 371.0 ± 107.74 cm^3^ for intramural myomas, 108.8 ± 23.50 cm^3^ for submucosal myomas (Table 1), and 379.5 cm^3^ for adenomyosis (data not shown). The average treatment time for myoma was 29.6 min (range 11.5–57.3 min) and the treatment time for adenomyosis was 55 min.

**Table 1:** Treatment outcome and disease characteristics of each patient

Case	Fibroid type	Funaki type	Tumor volume (cm^3^)			Shrinkage (%)		SF-36 score		
			Pretreatment	1-Month follow-up	3-Month follow-up	1-Month follow-up	3-Month follow-up	Pretreatment	1-Month follow-up	3-Month follow-up
01	Subserosal	2	191.8	163.1	202.2	14.96	− 5.42	87	83	89
02	Subserosal	1	348.6	380.6	347.1	− 9.18	0.43	77	83	84
03	Subserosal	1	280.6	244.5	225.5	12.87	19.64	75	79	83
04	Intramural	3	525.6	298.2	216.2	43.26	58.87	81	83	83
05	Subserosal	1	272.5	266.3	229.5	2.28	15.78	70	75	77
06	Intramural	2	423.7	338.4	353.4	20.13	16.59	87	90	83
07	Submucosal	3	132.3	177.2	132.3	− 33.94	0.00	67	77	49
08	Submucosal	1	85.3	90.2	79.2	− 5.74	7.15	76	58	53
09	Intramural	2	163.7	163.7	163.7	0.00	0.00	75	60	74

SF-36: 36-Item Short Form Survey

At 3 months after treatment, the mean shrinkage ratio was 12.56 ± 6.49%. MRgHIFU treatment reduced fibroid size in seven patients (subserosal *n* = 3; intramural *n* = 2; submucosal *n* = 1; diffuse adenomyosis *n* = 1); one subserosal case experienced a slight increase of 5.42% in fibroid size. The mean extent of tumor shrinkage was 7.6 ± 6.01% in the subserosal myomas, 25.2 ± 17.53% in the intramural myomas, 3.6 ± 3.58% in the submucosal myomas (Table 2), and 5.5% for the adenomyosis case (data not shown), while the highest shrinkage ratio by Funaki classification was in type 3 fibroids, followed by type 1 and lastly type 2; some of the type 2 fibroids increased in volume after treatment (Table 3).

**Table 2:** Treatment outcomes summarized by fibroid type

	Subserosal myoma N = 4	Intramural myoma N = 3	Submucosal myoma N = 2
Tumor volume (cm^3^)			
Pretreatment	273.4 ± 32.10	371.0 ± 107.74	108.8 ± 23.50
1-month follow-up	263.6 ± 44.87	266.8 ± 52.82	133.7 ± 43.50
3-month follow-up	251.1 ± 32.57	244.4 ± 56.55	105.8 ± 26.55
Shrinkage (%)			
1-month follow-up	5.2 ± 5.55	21.1 ± 12.50	− 19.8 ± 14.10
3-month follow-up	7.6 ± 6.01	25.2 ± 17.53	3.6 ± 3.58
Total SF-36 score			
Pretreatment	77.2 ± 3.44	80.8 ± 3.37	71.7 ± 4.71
1-month follow-up	80.1 ± 2.08	77.6 ± 9.13	67.4 ± 9.83
3-month follow-up	83.2 ± 2.47	80.0 ± 3.18	51.0 ± 1.74

SF-36: 36-Item Short Form Survey

**Table 3:** Treatment outcomes summarized by Funaki type

	Funaki type 1 N = 4	Funaki type 2 N = 3	Funaki type 3 N = 2
Tumor volume (cm^3^)			
Pretreatment	246.8 ± 56.46	259.7 ± 82.38	329.0 ± 196.65
1-month follow-up	245.4 ± 59.72	221.7 ± 58.33	237.7 ± 60.50
3-month follow-up	220.3 ± 54.85	239.8 ± 57.89	174.3 ± 41.95
Shrinkage (%)			
1-month follow-up	0.1 ± 4.90	11.7 ± 6.04	4.7 ± 38.60
3-month follow-up	10.7 ± 4.32	3.7 ± 6.62	29.4 ± 29.44
Total SF-36 score			
Pretreatment	74.7 ± 1.53	82.7 ± 3.89	74.0 ± 6.96
1-month follow-up	73.6 ± 5.65	77.7 ± 9.15	80.2 ± 3.00
3-month follow-up	74.2 ± 7.32	81.9 ± 4.43	66.2 ± 16.97

SF-36: 36-Item Short Form Survey

Most patients showed stable or improved SF-36 scores over 3 months of follow-up. The mean SF-36 score was 77.2 ± 3.44 before treatment and 83.2 ± 2.47 at 3 months for the subserosal myoma cohort; corresponding values were 80.8 ± 3.37 and 80.0 ± 3.18, respectively, for the intramural myoma cases (Table 2). In contrast, SF-36 scores fell over time in submucosal myoma cases, from 71.7 ± 4.71 points at baseline to 51.0 ± 1.74 points at 3 months. SF-36 scores were stable over time for fibroids classified by Funaki type: the mean SF-36 score was 74.7 ± 1.53 before treatment and 74.2 ± 7.32 at 3 months for type 1; 82.7 ± 3.89 and 81.9 ± 4.43, respectively, for type 2; and 74.0 ± 6.69 and 66.2 ± 16.97, respectively, for type 3 (Table 3). The adenomyosis case experienced an improvement of 6 points in SF-36 score at 3 months (baseline: 63; 3 months: 69; data not shown). During the 3 months of follow-up, all participants completed the treatment without common complications or adverse effects, including skin burn and abdominal pain, abdominal wall edema, bladder wall injury, vaginal discharge, and sacral bone edema.

## Discussion

The application of HIFU as the treatment for uterine fibroids has been studied for many years and is now considered an appropriate alternative therapy for patients who do not respond to conservative treatments. Despite the non-invasive feature of HIFU, many limitations related to technique and mechanical design have prevented its widespread application [22, 28].

Current MRgHIFU treatment protocols suggest an ablation rate (i.e., nonperfused volume [NPV] ratio) of 70–80% to achieve the desired treatment outcomes [21, 29]. In our study, we complied with the TFDA’s safety considerations to produce an NPV ratio of under 50%, which led to a mean fibroid volume reduction rate of 12.56 ± 6.49% at 3 months of follow-up. The effectiveness of ArcBlate MRgHIFU seemed not to be inferior to that of other MRgHIFU systems, which demonstrated mean fibroid volume shrinkage rates of 12.6–18.1% at 3 months [30, 31].

A higher NPV ratio was found to be correlated with greater tumor volume shrinkage. Keserci et al. discovered that if the NPV achieved a ratio of at least 90%, the mean fibroid reduction ratio would be as high as 54 ± 13% [31]. A report of 80 patients with leiomyoma suggested that a larger NPV ratio would result in greater shrinkage and improved relief of symptoms [32]. Although we performed an NPV ratio of under 50% per TFDA recommendations, future research is warranted to find a best NPV ratio with balanced therapeutic efficacy and safety.

**Fig. 2:**
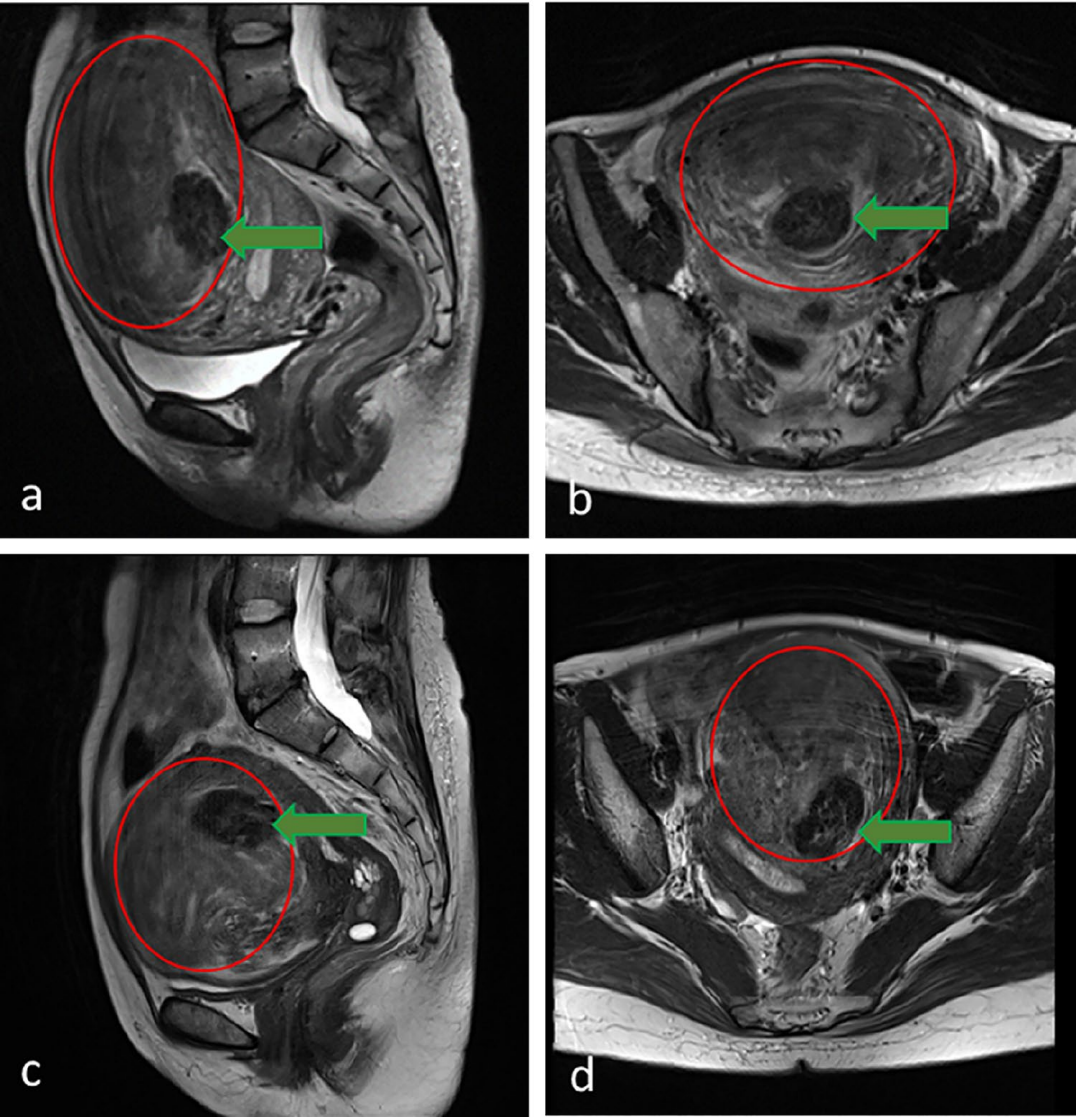
T2-weighted MR images of case no. 4, a 49-year-old woman with Funaki type 1 within the Funaki type 3 fibroid. Images include pretreatment (**a**, **b**) and post-treatment three-month follow-up (**c**, **d**). The pre-treated volume of fibroid was 525.6 cm^3^, and the post-treated volume was 216.2 cm^3^, with a shrinkage rate of 58.87%. Sagittal view: (**a**, **c**); axial view: (**b**, **d**). Red circles were the area of Funaki type 3 uterine fibroid; green arrows were the area of Funaki type 1 uterine fibroid

Regardless of the low NPV ratio, one intramural case in our study demonstrated an astonishing mass reduction of 58.8% at 3 months after the treatment. According to the literature, treating Funaki type 3 fibroid tissue by HIFU is difficult because of high vascularization which results in decreasing therapeutic effects of heating [23, 33, 34], yet our intramural case was identified to be Funaki type 3. During treatment, her physician found a solid area with fibroid tissue similar to Funaki type 1 within the Funaki type 3 fibroid (Fig. 2). The treatment plan was to focus mainly on the type 1-like tissue, yet an overall mass reduction was observed. The treatment effect may be attributed to the accelerated metabolism of ablated tissue by rich vascularity, which may have influenced the surrounding mass. Despite the decreased estrogen level in this patient (49 years) who approached menopause may also have influenced the treatment outcome [35], our result still suggests a new approach toward a more efficient treatment of mixed fibroid presentation.

No real-time monitoring in thermal damage in tissue was one of the limitations in ultrasonography-guided HIFU therapy [36], while it could be resolved under MR guidance. Besides, patients having pelvic endometriosis, adhesions locating between the uterus and bowel, or > 10-mm abdominal surgical scar are not recommended to receive HIFU therapy [37]. However, one patient with adenomyosis in our study showed a lower shrinkage rate after 3 months than another study (our study vs. another study: 5.5% vs. 46.3%) [38]. Possible reasons for different shrinkage rates might include the treated volume limited in the current study (no more than 50%), different lesion type (current study vs. Jeng et al.’s study: diffuse vs. focal/regional), and study design (prospective vs. retrospective). However, it should be interpreted with caution due to the small sample size.

Moreover, we observed that the fibroid size in some patients became larger at 1-month follow-up after HIFU therapy. Transient inflammatory or congestive response caused by HIFU possibly leads to increase the fibroid size at acute phase, and size shrinkage was observed in the 3-month follow-up. A longitudinal study to assess the inflammatory response to HIFU is warranted.

[39] Considering the treatment outcome among patients by Funaki classification, quality of life scores in the type 3 cohort seemed to decrease slightly after the treatment, despite these fibroids demonstrating considerable reductions in size at 3 months (mean shrinkage: 29.4% ± 29.44). In contrast, quality of life for the Funaki type 1 and 2 cohorts was much more stable, despite lacking remarkable reductions in tumor size (mean shrinkage: type 1, 10.7% ± 4.32; type 2, 3.7% ± 6.62). The better quality of life score of Funaki type 1 and 2 groups before treatment may have influenced physical and mental health status after treatment. Also, the results may be biased by the small sample size. Further large-scale investigations are warranted to validate our findings.

The most common adverse effect of HIFU treatment is skin burn [30, 39]; none of our patients reported this effect through 3 months of follow-up. Use of the soft water bag may have reduced the risks of skin burn by fitting the abdominal contour, binding with the HIFU transducer, and moving with the transducer to ensure direct skin contact. Another potential protective mechanism against skin burns is that putting the patient in the supine position allows for accurate monitoring of irregularities on the abdominal surface.

Much research has shown that rectal and bladder filling are very useful practices for avoiding adverse effects and for mitigating bowel-related side effects after MRgHIFU treatment [40, 41]. Except for the fibroid location of patients be close to the intestinal tract, we did not follow these practices in our study, because they can be uncomfortable for patients. In addition, endometrial ablation has been used to treat submucosal fibroids [42] but it may cause severe endometrial impairment and reduce possibilities of future pregnancy [43]. We therefore did not ablate the endometrium during treatment, to prevent any adverse consequences regarding future pregnancy.

## Conclusion

Although this was a feasibility study in a small population, the effectiveness and safety of ArcBlate MRgHIFU were investigated in a wide range of patient characteristics. Our system demonstrated clinical effects similar to those demonstrated by other MRgHIFU systems and it proved to be safe. ArcBlate MRgHIFU has the potential to be an alternative, non-invasive treatment option for uterine benign tumors.

## Data Availability

The data shown in this study are available on request from the corresponding author.
